# Global health experiences of U.S. Physicians: a mixed methods survey of clinician-researchers and health policy leaders

**DOI:** 10.1186/1744-8603-9-19

**Published:** 2013-05-11

**Authors:** S Ryan Greysen, Adam K Richards, Sidney Coupet, Mayur M Desai, Aasim I Padela

**Affiliations:** 1 Division of Hospital Medicine, University of California San Francisco, 533 Parnassus Avenue, Suite U112, Box 0131, San Francisco, CA 94143-0131, USA; 2 Department of General Internal Medicine and Health Services Research, University of California, Los Angeles, USA; 3 Robert Wood Johnson Foundation – Clinical Scholars Program, University of Michigan, Ann Arbor, USA; 4 Robert Wood Johnson Foundation – Clinical Scholars Program, Yale University, New Haven, USA; 5 Division of Chronic Disease Epidemiology, Yale School of Public Health, New Haven, USA; 6 Sections of Emergency Medicine and General Internal Medicine, Department of Medicine, University of Chicago, Chicago, USA

**Keywords:** Global health, International medicine, Health policy, Career development

## Abstract

**Background:**

Interest and participation in global health activities among U.S. medical trainees has increased sharply in recent decades, yet the global health activities of physicians who have completed residency training remain understudied. Our objectives were to assess associations between individual characteristics and patterns of post-residency global health activities across the domains of health policy, education, and research.

**Methods:**

Cross-sectional, mixed methods national survey of 521 physicians with formal training in clinical and health services research and policy leadership. Main measures were post-residency global health activity and characteristics of this activity (location, funding, products, and perceived synergy with domestic activities).

**Results:**

Most respondents (73%) hold faculty appointments across 84 U.S. medical schools and a strong plurality (46%) are trained in internal medicine. Nearly half of all respondents (44%) reported some global health activity after residency; however, the majority of this group (73%) reported spending ≤10% of professional time on global health in the past year. Among those active in global health, the majority (78%) reported receiving some funding for their global health activities, and most (83%) reported at least one scholarly, educational, or other product resulting from this work. Many respondents perceived synergies between domestic and global health activities, with 85% agreeing with the statement that their global health activities had enhanced the quality of their domestic work and increased their level of involvement with vulnerable populations, health policy advocacy, or research on the social determinants of health. Despite these perceived synergies, qualitative data from in-depth interviews revealed personal and institutional barriers to sustained global health involvement, including work-family balance and a lack of specific avenues for career development in global health.

**Conclusions:**

Post-residency global health activity is common in this diverse, multi-specialty group of physicians. Although those with global health experience describe synergies with their domestic work, the lack of established career development pathways may limit the benefits of this synergy for individuals and their institutions.

## Introduction

Interest in global health (GH) among physicians in training has steadily increased over the last three decades. U.S. Academic Medical Centers (AMCs) [1,2], the National Institutes of Health (NIH) [3], U.S. Government (USG) [4], and philanthropic foundations [5] have responded to this growing demand by investing in GH training. [6] In 1978, 6% of U.S. medical graduates reported experiences in GH, but today more than 25% participate in GH activities during medical school, and 66% of physicians entering the medical profession plan to participate in GH work during their career [7]. While a growing literature describes the advantages and challenges of GH experience during medical school [8-10], and residency [11,12], little is known about patterns of GH activity among physicians who have completed their medical training or on the challenges they perceive in carrying out this work.

Although information about the post-training GH experiences of “typical” physicians engaged in clinical practice abroad could be used to maximize contributions of these physicians in clinical settings outside the U.S., our survey focuses specifically on the GH experiences of U.S. physicians with training in health education, research and policy for several reasons. First, participation of physicians with training in these areas is complementary to clinical practice and crucial to drive systems change within key institutions such as NIH, USG, and AMCs. Data on the GH involvement of this group can help align priorities within these institutions, the aims of more traditionally GH-focused funders and stakeholders, and the GH interests of the broader U.S. physician workforce [13,14]. Second, better understanding of challenges to career development in GH for physician-leaders at AMCs specifically can be translated into strategies to recruit and retain globally-engaged faculty who will promote synergy between domestic and GH activities for the next generation of physicians across a wide range of specialties [13,15]. Third, physicians with clinical specialty training may be well-positioned for GH leadership roles within medical schools, hospitals, and other institutions [16-18]. Similarly, physicians with specialized training related to health education, research and policy are uniquely positioned to contribute to previously neglected but currently crucial global health priorities including: public health education, health system strengthening, quality improvement, non-communicable disease control, community-driven outcomes research, social determinants of health, health policy, cost effectiveness, and leadership development.

Accordingly, we conducted a mixed-methods study of 521 physician-leaders, including 378 academic physicians at 84 U.S. medical schools, who had received inter-disciplinary health services research training through the Robert Wood Johnson Foundation Clinical Scholars Program (RWJF CSP) over the last 39 years [19]. Our quantitative survey examined relationships between individual demographic and specialty characteristics and patterns of involvement in GH, and assessed perceptions about synergies between domestic and GH activities. Qualitative interviews further explored perceived synergies, barriers and facilitators of GH work in multiple institutional settings. Our overall objectives were to describe the characteristics of these globally-engaged physician-leaders, patterns of their post-residency global health activities, and challenges to continued involvement across domains of their expertise in health policy, education, and research.

## Methods

### Study design and sample

We conducted an online cross-sectional survey of current and former fellows of the RWJF CSP and interviewed a purposive sample of respondents. Several factors make the Clinical Scholars Program an ideal cohort to study GH activities of physician-leaders in health policy, education, and research. First, the RWJF CSP has thoroughly transformed the health and healthcare of Americans. Alumni include 8 medical school deans, 51 division or department chairs, 28 Institute of Medicine members, and many high-level administrators in public service (e.g. Surgeon General, Department of Health and Human Services Assistant Secretary) and healthcare systems (e.g. hospital chief executive officers) [19]. Second, the fellowship trains physicians in skills that complement emerging priorities in GH, such as clinical epidemiology, health system strengthening and health services research, social determinants of health, clinical and public health education, health policy, and leadership. Third, the RWJF CSP has been in continuous operation for nearly 40 years, making it the oldest and largest program dedicated to clinical research and health policy leadership in the U.S [20]. Although this group may not be representative of ”typical” U.S. physicians, it was not our intention to survey physicians whose training and career were exclusively focused on clinical practice. Our objectives were to understand the GH experiences of physician-leaders in health policy, education, and research. The uniqueness of this cohort notwithstanding, graduates of the RWJF CSP are highly diverse in terms of age, gender, race/ethnicity and clinical practice, with 24 specialties represented.

### Study instruments: survey tool and interview guide

We designed an online survey instrument (hosted by SurveyMonkey.com) informed by the GH experiences of the investigators and input from the RWJF National Program Office and the National Advisory Committee for the CSP [21]. The survey instrument contained 24 multiple-choice questions on demographics, GH experiences, funding sources, end products, and views about synergies between domestic and GH activities. We defined GH experience broadly as “any research, clinical, educational or health policy activity that engages or directly impacts populations, stakeholders or systems outside the United States.” [22] The survey instrument was pilot-tested with a diverse group of health services researchers who had GH experiences to assure content appropriateness and face validity.

In addition, we designed a qualitative interview guide based on a literature review of the challenges and experiences of physicians engaged in GH work and discussions focused on GH at national meetings of the RWJF Clinical Scholars Program. Conceptual domains to be explored during in-depth interviews included motivations for and scope of GH activities, synergies between GH priorities and domestic engagements, and perceived facilitators of and barriers to GH career development.

### Data collection

Quantitative survey: We distributed a link to the online survey to all fellows and graduates with email addresses in the RWJF CSP alumni database. Of the 1,147 fellows and graduates listed in October 2010, we excluded those without email addresses (n=190) and those whose emails were non-functional (n=181). Thus, our initial sampling frame contained 776 physicians. After receiving up to 3 reminder emails at 4-week intervals, a total of 521 (67%) completed the survey.

Qualitative interviews: We divided the 229 (44%) survey participants who indicated a willingness to participate in follow-up interviews into four categories according to reported level of GH activity: prior GH activity but no current engagement; planned GH activity in the future but none currently; moderate level of current GH activity; and high level of current GH activity. Using maximum variation purposeful sampling, [23] we selected participants from each category and two members of the research team (AKR, AIP) conducted individual interviews between June and July 2011 until thematic saturation was reached on the motivations for seeking GH experiences and on the types of activities participants engaged in. Interviews lasted approximately 45 minutes and were conducted using a secure telephone line with verbatim audio-recording.

### Analysis

Quantitative analysis of survey responses included simple frequency distributions to describe characteristics of the entire sample, and bivariate statistics (chi-squared tests) to evaluate unadjusted associations between sample characteristics and level of GH experience. Since all respondents had already completed residency training but some were still in the fellowship program, we dichotomized GH experience for the entire sample to any GH experience vs. no experience during/since fellowship. We then further categorized the group with any GH experience as no time spent on GH in last year, up to 10% time spent on GH in last year, or over 10% time spent on GH in last year. To better characterize the 229 respondents with any GH experience during/since fellowship, we created mutually exclusive categories of experience level: “low” (1 or 2 GH projects during/since fellowship and less than or equal to 10% time in last year), “moderate” (3 to 5 projects during/since fellowship or 11% to 30% time in last year), and “high” (more than 5 projects ever or more than 30% time in last year). All analyses were performed in STATA 11.2 (College Station, TX).

Content analysis of qualitative interviews (n=15) utilized a team-based, crystallization-immersion approach [24]. Three authors independently read the transcripts and identified emerging themes within each interview, and presented a thematic summary of interviews and illustrative quotes during team meetings. Disagreements were resolved through negotiated team consensus. The entire team met to select representative quotations that illustrated or extended quantitative survey findings.

## Results

Among the 521 participants who responded to the online survey, 60% were male; 46% had internal medicine or medicine subspecialty training; 73% indicated university as their primary professional setting; and 52% indicated research as their primary professional activity (Table 1). Additionally, 46% reported fluency in at least one language beyond English, and 32% reported that a first-degree relative had been born outside the U.S. (data not shown).

**Table 1:** Characteristics and global health experience during/since fellowship of the overall sample (n=521), overall and by intensity of recent global health engagement

			**Reporting global health experience during/since fellowship (n=229)**					
	**Total sample**		**Overall**		**Time dedicated to GH in the past year**			
					**None**	**1-10% time**	**>10% time**	
	**N**	**%**	**%**	**p-valueφ**		**Row %**		**p-valueΘ**
	**521**	**100%**	**44%**		**20%**	**53%**	**27%**	
**Demographics**								
**Graduation Year**				**<0.01**				0.25
Before 1980	75	14%	64		21	60	19	
1980-1989	101	19%	61		26	52	23	
1990-1999	111	21%	53		19	44	37	
2000-2009	161	31%	29		15	61	24	
After 2009	73	14%	19		7	50	43	
**Gender**				**<0.01**				0.47
Female	208	40%	36		24	50	26	
Male	313	60%	50		17	55	28	
**Professional characteristics**								
**Clinical Specialty**				0.25				0.96
Internal medicine and	241	46%	48		18	54	28	
subspecialties								
Pediatrics and subspecialties	97	19%	44		19	56	26	
Surgery and subspecialties	51	10%	37		21	58	21	
All other specialties	132	25%	39		24	47	29	
**Primary Professional Setting**				**0.02**				0.23
University	378	73%	41		21	57	22	
Public sector	50	10%	50		24	48	28	
Private sector	54	10%	44		13	46	42	
Foundation, NGO, or other	39	8%	67		15	42	42	
**Primary Professional Activity**				**<0.01**				0.26
Research	271	52%	41		20	52	29	
Clinical	72	14%	28		20	65	15	
Education	45	9%	58		31	58	12	
Administration, policy, other	133	26%	53		15	51	34	

φ p-value for global health experience since fellowship (vs. no experience).

Θ p-value for professional time allocated to global health among those with global health experience during/since fellowship.

### Characteristics of physicians with global health experience

Overall, 44% of all respondents in our study reported having at least some GH experience either during or after their RWJF Clinical Scholar Program training. Among this group of 229 respondents, 107 (53%) reported GH experience totaling 1-10% of their total professional time in the last year, while only 62 (27%) reported allocating more than 10% of their time to GH work in the previous year (Table 1). Although GH experience was positively associated with time since completion of fellowship, the amount of time dedicated to GH efforts in the last year among those with GH experience was generally similar across cohorts. Similarly, although male participants were more likely than female participants to report any GH experience during/since fellowship (50% of men vs. 36% of women, p<0.01), men and women with GH experience reported similar time spent on GH in the last year (p=0.47) (Table 1).

GH experience did not appear to differ by clinical specialty (p=0.25), but GH experience was less commonly reported by respondents who identified research (41%) or clinical work (28%) as their primary professional activity, compared to respondents who identified education (58%) or administration, policy, or “other” as their primary role (53%) (p<0.01). Regarding primary professional setting, the proportion of respondents with GH experience among those who indicated university as their primary setting (41%) was less than that for public (50%), private (44%), and foundation, Non-Governmental Organization (NGO), or “other” (67%) (Table 1).

### Patterns of global health experience

**Figure 1:**
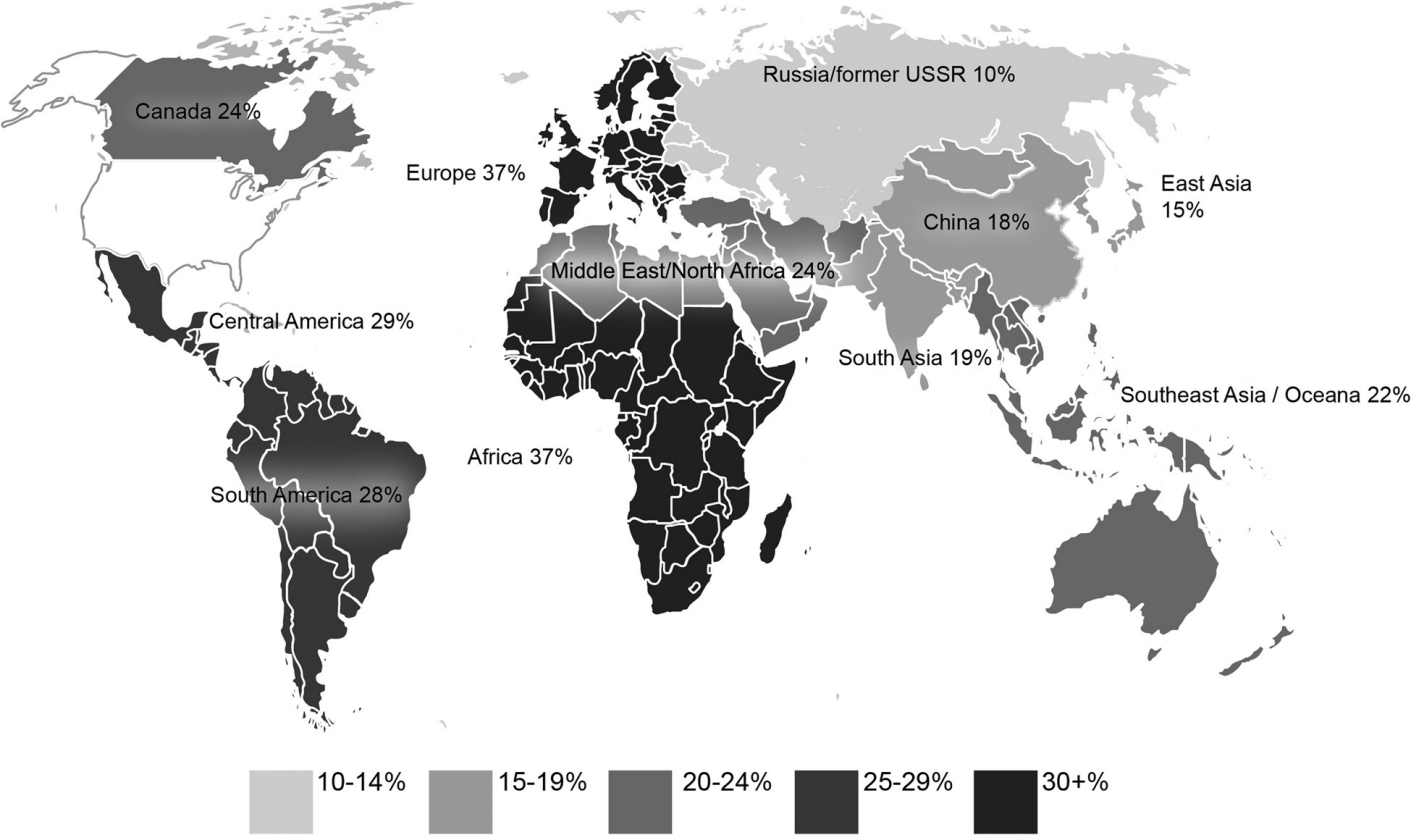
Geographic Location of GH Activity (Percent of Respondents).

Our sample included respondents with GH experience in every major geographic region of the world. The most frequently engaged regions were Africa (37%), Europe (37%), Central America (29%), and South America (28%) (Figure 1). The majority of GH-engaged respondents (83%) reported at least one product from their GH activities (Table 2). The most common products were media interviews, internet-only reports, or teaching materials (57%), followed by peer-reviewed publications (47%). In addition, significant associations were found between higher GH experience and each of the product types. Most respondents (78%) reported having received formal funding for their GH work, and significant associations were seen between increasing GH experience and proportion of respondents receiving funding in every funding category except International Monetary Fund (IMF), NGO, or other organization (Table 2). Qualitative data revealed several concerns and challenges related to initiating or sustaining global health activities as shown below:

**Table 2:** Characteristics of global health work of respondents reporting global health experience during/since fellowship (n=229), overall and by level of reported experience

**Characteristic**	**Overall**			Level of Global Health Experience*		
				**Low**	**Moderate**	**High**
	**% Reporting product, funder, population, or disease focus**					
	**N**	**%**	**%**	**%**	**%**	**p-value**
**Product Type**						
Peer-reviewed publication	108	47%	29%	57%	69%	**<0.01**
Government or NGO report	81	35%	17	41	61	**<0.01**
Book or monograph	46	20%	5	17	48	**<0.01**
Media, web-only, or teaching material	131	57%	48	55	75	**<0.01**
Any above product	190	83%	73	90	94	**<0.01**
**Funder of Global Health Work**						
NIH, CDC, or other US government agency	82	36%	22	43	52	**<0.01**
Other (non-US) government	62	27%	22	22	39	**0.04**
World Health Organization	33	14%	7	19	23	**<0.01**
Philanthropy	100	44%	35	45	58	**0.01**
IMF, NGO, or other organization	43	19%	15	17	27	0.16
Any above funding	179	78%	67	86	89	**<0.01**
**Population Focus of Global Health Work**						
General population	101	44%	38	38	59	**0.02**
Health workforce (clinicians)	104	45%	38	47	56	0.07
Women or children	76	33%	31	22	47	**0.01**
HIV/AIDS	43	19%	12	17	31	**<0.01**
Other population focus**	37	16%	16	10	22	0.22
**Disease Focus of Global Health Work**						
Infectious diseases	85	37%	32	38	45	0.21
Chronic diseases (non-infectious, cancer)	82	36%	29	38	45	0.09
Mental health or neurologic	43	19%	16	26	17	0.27
Reproductive health	36	16%	15	12	20	0.44
Injury	31	14%	9	9	23	**0.02**
Nutrition	24	10%	10	5	16	0.17
Surgical	12	5%	7	3	5	0.68
Environmental / occupational	11	5%	3	3	9	0.13

* Low/Moderate/High level of GH experience defined by combination of 1) total number of GH products produced in career to date and 2) percentage of time dedicated to GH work in the past year. See text for details.

** Other population focus = displaced persons/refugees, racial/ethnic minorities, or elderly.

Themes from Qualitative Analysis

I. Challenges of doing global health work

a. Family and personal considerations

a. Career development and other professional considerations

a. Funding challenges

II. Benefits of doing global health work

a. Developing a broader “systems perspective” and appreciation for social determinates of health

a. Developing a broader set of cross-cultural skills and greater desire to work with underserved domestic populations

### Challenges posed by global health activities

Many respondents articulated concerns about obligations to their family that could interrupt plans to participate in GH work. As one female physician explained,

“It’s just hard to figure out how to marry [global health work] with having a life that’s sustainable. I was supposed to go to Haiti…[but] my son got hospitalized and I had to cancel…anytime I’ve tried to have those kind of experiences in the last few years, it sort of falls apart because of family issues. One of my very best friends from high school works for [an international nonprofit]…she’s married and it’s a huge toll…it’s a complex issue I think especially for women.” (Junior faculty, emergency medicine)

### Family obligations created challenges for men as well

“I had a family at that time, my wife and a couple of kids. A few months abroad to do design and do some research probably would not have been in the cards.” (Senior government service officer, preventative medicine)

Several respondents also commented on the role of structural challenges to career development for physicians wanting to dedicate significant amounts of time to GH work. One respondent noted that lack of familiarity with GH activities among employers can be problematic within a variety of institutional settings:

“There aren’t as many people doing global health work and institutions have built-in barriers that you’re not even aware of until you [encounter them]. Administratively, your department chair, your division head … some of them are much better at understanding what it is that you do and [they] value it but others… less so. Part of it depends on [their] personal experiences but [there is a] general tendency to say, ‘you’re at a state institution in California, so what you should do is to focus in on Californians.’” (Senior faculty, pediatric research and education)

Another respondent with leadership experience in academic medicine commented more specifically on the lack of established GH pathways in academic medicine:

“You don’t want to be a square peg in a round hole and [you often encounter] difficulty with folks understanding what you’re doing, problems with promotion, tenure and things of that sort. The career trajectories in this area are still in the formative stages. The mentorship is still evolving. The kind of grants and support you can get and journals you can publish in, they’re all still evolving.” (Senior faculty, internal medicine and sociology)

Several respondents described specific challenges in obtaining funding for GH work during in- depth interviews. One respondent described challenges related to obtaining funding for collaborative work in higher-income countries:

“I think it’s a little bit easier to get funding to work in developing countries [but for] developed countries like Japan, it’s very difficult to get funding. I would be very scrappy, you know, bark up every tree to see what funding might be available…[but] it is very difficult to get funding to do [this kind of] international work.” (Senior faculty, pediatric research and education)

Another respondent described broader challenges in obtaining funding for GH work and an inconsistency between funding priorities in two different areas within the same institution:

“I think one of the biggest challenges [to global health careers] is clearly funding… The CDC, as an example, has injury control in their mandate for research [but] explicitly excludes any research being done out of the country. It doesn’t mean that they don’t have some interest in global injury control, but they certainly don’t see it as part of their mission in the same way they see global infectious disease as being part of their mission” (Senior faculty, pediatric research)

### Synergies between domestic and global health activities

Among respondents with GH experience, there was strong agreement that their GH activities are synergistic with their domestic work: overall 85% of respondents agreed that their GH activities had enhanced the quality of their domestic work; and agreement with this statement was more common among respondents with moderate or high level of experience (91-92%) than among respondents with low level of experience (78%) (Table 3). Qualitative data from participant interviews contrasts the benefits of these synergies with reported challenges of GH work ee section “Themes from Qualitative Analysis” above). One participant explained that their GH experiences fundamentally changed their approach to their career:

“I think [that it] opened up my view of healthcare through a bunch of international experiences. These fundamentally guided and directed me to become more of a generalist than a specialist, to consider the social sciences as extremely important for all aspects of health care delivery, education, and research.” (Senior faculty, internal medicine and sociology)

**Table 3:** Global health experience and domestic health priorities among respondents reporting global health experience during/since fellowship (n=223), overall and by level of reported experience

**Statement**		**Overall**			Level of GH experience*		
					**Low**	**Moderate**	**High**
		**% Responding “Agree or Strongly Agree”**					
	**Valid Responses**	**n**	**%**	**%**	**%**	**%**	**p-value**
My domestic (U.S.-only) work has enhanced the quality of my global health work.	223	204	92%	88%	98%	92%	0.07
My global health work has enhanced the quality of my domestic (U.S.-only) work.	223	190	85	78	91	92	**0.02**
My global health experience has increased my level of involvement with:							
conducting research, clinical care, or influencing policy for **underserved populations**	219	138	63	64	64	60	0.85
engaging in efforts to promote change in **health policy**	220	139	63	62	69	60	0.55
addressing **social determinants of health **through research and clinical care	117	136	63	59	64	68	0.48
becoming **involved in my community**	217	108	50	49	48	53	0.81
conducting research, clinical care, or influencing policy for **health of immigrants **to the United States	215	89	41	36	47	46	0.29

* Low/Moderate/High level of GH experience defined by combination of 1) total number of GH products produced in career to date and 2) percentage of time dedicated to GH work in the past year.

Most respondents with GH experience agreed that their GH activities had contributed to greater community involvement, engagement of underserved patients, and promotion of health policy changes, however; agreement in these domains was not associated with level of GH experience (p>0.25 for each domain, Table 3). One respondent described the effect of GH activities on seeing a “larger context” for clinical practice and health policy at home:

“I clearly learned a lot from global experiences that have helped to influence my work here, working with underserved families and really a commitment to behavior change in the larger context, outside of just the physician’s office, about what can we do for policies and what can we do that’s effective to change behavior.” (Senior faculty, pediatric research)

Another respondent explained how GH work with refugee populations provided insights for him into the social barriers to caring for highly vulnerable populations at home:

“It was very clear to me that there was tremendous crossover in the approach to both underserved US populations as well as international populations…there was as much social distance between me and a homeless guy who had been living on a street for 30 years as there was between me and a Cambodian refugee.” (Senior internist and Executive Director of global health non-profit)

Finally, one training program director highlighted both the benefits of these synergies and challenges to building academic careers from these experiences. Specifically, this respondent highlighted the need to develop new career “paths” and institutional resources to support these careers:

“For the residents who go over to Africa… when they come back what kind of path do they have? It’s pretty significant to have given two or three years of service but…academically that counts for nothing. We have to put in place [systems] to make this a reasonable choice…. I don’t want people getting stuck because they did something really good for humanity but it really didn’t help them career-wise in the end. We’re trying to think about what kind of institutional resources we can use… so that when people come back, they can have a good job… a place to come back and get reintegrated.” (Senior faculty, pediatric research)

## Discussion

This study of global health activities in a national, multi-specialty sample of U.S. physician-leaders holds implications for academic and other institutions providing careers for physicians with advanced training in research and policy. Nearly half (44%) of our respondents have participated in GH work during or after completion of residency training. Most respondents (73%), however, reported spending 10% or less of their professional time on GH activities within the past year. This suggests that many physicians in our sample participate in GH activities on a part-time basis, and that such activities do not represent the primary focus of their careers. Despite this part-time involvement, many of these physicians successfully obtained funding for their GH activities, created dissemination products, and perceived robust synergies between their GH experience and their domestic work. While the participants in our study are a select group of physician-leaders with specialized research training, many of them reported deliverables other than peer-reviewed publications as outputs of their GH work, such as web-based material, and NGO or government reports. This suggests that even a highly-trained group of physician-leaders find it necessary to adapt their skillsets to generate products that are relevant to GH stakeholders.

Indeed, this select cohort of physician-leaders with unique career development resources from their participation in the RWJF Clinical Scholars program still encountered significant challenges and barriers to their GH activities; these barriers may be even more important to address for physicians who do not have the same resources and skillsets to deploy as the physicians in our cohort. First, we observed a significant gender gap among respondents reporting GH experience. This suggests that special career development resources may be required to ensure gender equity in GH opportunities, perhaps as part of larger efforts to address barriers to gender equity in academia and other leadership settings in medicine [25-27]. Second, we found that respondents who reported university as their primary professional setting were less likely to report GH experience than respondents in other institutional settings. Academic programs could reverse this trend through focused efforts to promote career development for faculty engaged in GH as part of broader efforts to minimize faculty attrition [28]. Indeed, opportunities for GH experience have become a definitive advantage for competitive residency programs and could yield similar dividends for faculty search committees [29,30]. Our findings also suggest that collaborative relationships with other GH stakeholders, such as government agencies, foundations, and international NGOs, could enhance faculty and program development at academic medical centers [31]. Respondents from a broad range of clinical specialties appeared similarly committed to GH in our sample. This suggests that global engagement can play an important role in supporting inter-disciplinary and team-based educational and training efforts at medical schools in the U.S. and abroad [32].

Finally, we found that the majority of physicians surveyed indicated that their global and domestic health work was highly synergistic. This is perhaps our most encouraging finding, as it suggests that U.S. physicians have heard recent calls to engage in GH work [33-35] and find this work complementary to their professional responsibilities at home in the U.S. Nonetheless, the eagerness of U.S. physicians to be more involved in GH activities also underscores important questions about competencies in GH [36]. Specific challenges include how to standardize GH training experiences [37] and respond to the growing demand for GH education; [38,39] how to professionalize a core humanitarian assistance workforce; [40] and how to transcend cultural competence to develop “transnational” competence [41]. Ultimately, academic medical centers and other drivers of U.S. health policy will need to address these issues not only due to the increased involvement in GH by U.S. physicians but also because of increasing global attention to the social accountability of medical education [42,43].

Our findings should be viewed in light of several limitations. First, we sampled physicians from an alumni database of the RWJF Clinical Scholars Program; physicians who have not participated in this program may hold different experiences and views. Other cohorts of physicians trained in education, research and policy could be more likely to report GH experience than ours given that the RWJF-CSP is a domestically-focused program without specific emphasis on global health. Nonetheless, it should be recognized that the vast majority of U.S. training programs from medical school to residency and fellowship focus on domestic issues (whether clinical, research or otherwise) but interest in GH continues to rise among most graduates despite this focus. Second, we did not assess timing of exposure and cannot comment on whether global health work inspired or evolved out of pre-existing professional interests, such as a passion for social determinants or improving the health of under-served populations. A third limitation, common to most survey-based studies, is that questions and multiple-choice responses do not fully capture the range and richness of respondents’ GH experience. To address this limitation we included a qualitative component to our project and explored nuances of experience among a purposeful sample of respondents. Fourth, responses to survey and interview questions are inevitably subject to recall bias. Finally, recent data are lacking to provide comparisons for our findings related to the GH experiences of US physicians. In 1984 Baker et al. conducted a survey of 1,267 organizations likely to hire health professionals to work internationally and estimated that 1,417 out of 450,000 physicians (0.32%) in the United States were working in international health [44]. Although the proportion of our cohort engaged in GH (44%) was over two orders of magnitude higher; it is not clear whether our estimate should be considered “high” or “low”, given our specific focus on physician-leaders with training in health education, research and policy, and the tectonic secular changes in professional medicine over the past thirty years. Additional study is needed to inform discussions about the frequency, focus, appropriateness and value of GH activity by U.S. physicians in general, and of specific groups of physicians with distinct skill sets, such as those trained in tropical medicine.

## Conclusions

In summary, we found that post-residency global health activity is common in a diverse, multi-specialty group of physicians with leadership and research training. Most physicians in our study engaged in GH activities on a part-time basis, but were often able to secure funding, create deliverable products from their work, and frequently found their global and domestic health activities to be highly synergistic. However, the lack of established career development pathways may limit the benefits of this synergy for individuals and their institutions. U.S. academic medical centers and other institutions that employ a substantial number of physicians engaged in GH should consider creating career development programs for GH to ensure continued and equitable involvement as well as to maximize their contributions to improving health worldwide.

## Competing interests

The authors declare that they have no competing interests.

## Authors’ contributions

SRG, AKR, and AIP conceived and designed the study together. SRG, AKR, SC, MMD, and AIP performed analysis and interpretation of data together. SRG wrote the first draft of the manuscript and all authors (SRG, AKR, SC, MMD, and AIP) contributed to manuscript revision and approved the final manuscript.
